# A Rare Case of Acute Myocardial Infarction due to Coronary Artery Dissection and Heparin-Induced Thrombocytopenia

**DOI:** 10.1155/2012/196020

**Published:** 2012-06-06

**Authors:** Michael G. Fradley, Douglas E. Drachman

**Affiliations:** Division of Cardiology, Department of Medicine, MA General Hospital, Boston, MA 02114, USA

## Abstract

Although both coronary artery dissection and heparin-induced thrombocytopenia may provoke myocardial infarction, it is extremely rare for both conditions to develop simultaneously in a single patient. We report a case of a 69-year-old woman who sustained a head-on motor vehicle accident with associated chest trauma. During a subsequent hospitalization, she was exposed to subcutaneous heparin and developed significant thrombocytopenia. Shortly thereafter, she re-presented with an acute myocardial infarction. Coronary angiography revealed a spiral dissection with superimposed thrombosis within the right coronary artery, while laboratory testing confirmed the diagnosis of heparin induced thrombocytopenia. She was treated with catheter-based thrombectomy and adjunctive direct thrombin inhibitor therapy, followed by three months of systemic anticoagulation with warfarin. To our knowledge, this represents the first published case of a native vessel myocardial infarction due to the combination of coronary artery dissection and heparin-induced thrombocytopenia.

## 1. Introduction

 Myocardial infarction typically occurs in patients with underlying atherosclerotic coronary artery disease. Other less common conditions may also lead to myocardial infarction even in the absence of atherosclerosis, however. The following case report illustrates an unusual etiologic mechanism for acute myocardial infarction, involving cardiac trauma with subsequent coronary dissection and endothelial disruption, followed by thrombosis in the context of heparin-induced thrombocytopenia.

## 2. Case Report

 A 69-year-old woman with a past medical history of hypertension presented to her primary care physician's office one day after a head-on motor vehicle accident. She had been wearing a seat-belt, and the airbag had deployed. Although paramedics had been called to the scene, the patient refused transport to the emergency room. A persistent, mild burning sensation in the chest prompted her to seek medical attention the following day. At that time, the physical examination was notable for contusion, with excoriation, swelling, and ecchymosis in the soft tissues overlying the left chest, sternum, and breast. The presentation was felt to be consistent with chest wall trauma. An EKG was not obtained. The patient was instructed to use acetaminophen and warm compresses for pain.

Several weeks later, the patient was hospitalized for a headache and acute hearing loss. The burning chest pain had resolved, and the chest contusion had markedly improved. During the five-day hospitalization, the patient received routine subcutaneous unfractionated heparin, deep venous thrombosis prophylaxis. Routine laboratory assessment on admission disclosed mild thrombocytosis, with a platelet count of 474 K/uL. Daily laboratory results were similar; however, on the day of discharge the platelet count had fallen to 174 K/uL. Although this represented a substantial decline of more than 50%, the absolute value remained within the normal reference range, and no additional workup was pursued.

**Figure 1:**
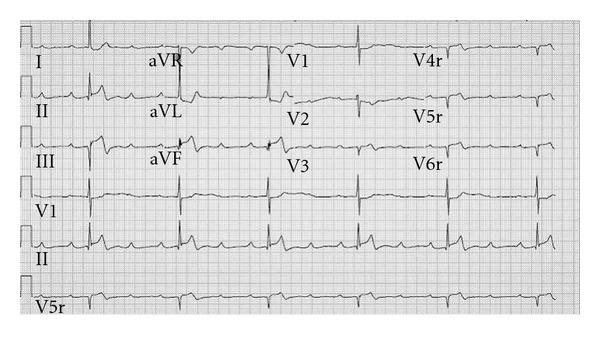
Right-sided surface electrocardiogram demonstrating 3rd-degree A-V block and a slow junctional escape rhythm, inferior ST segment elevations with reciprocal lateral ST segment depressions, inferior Q waves, prominent R wave in V1, and right-sided ST elevations in leads V4r–V6r suggesting acute right ventricular myocardial infarction.

**Figure 2:**
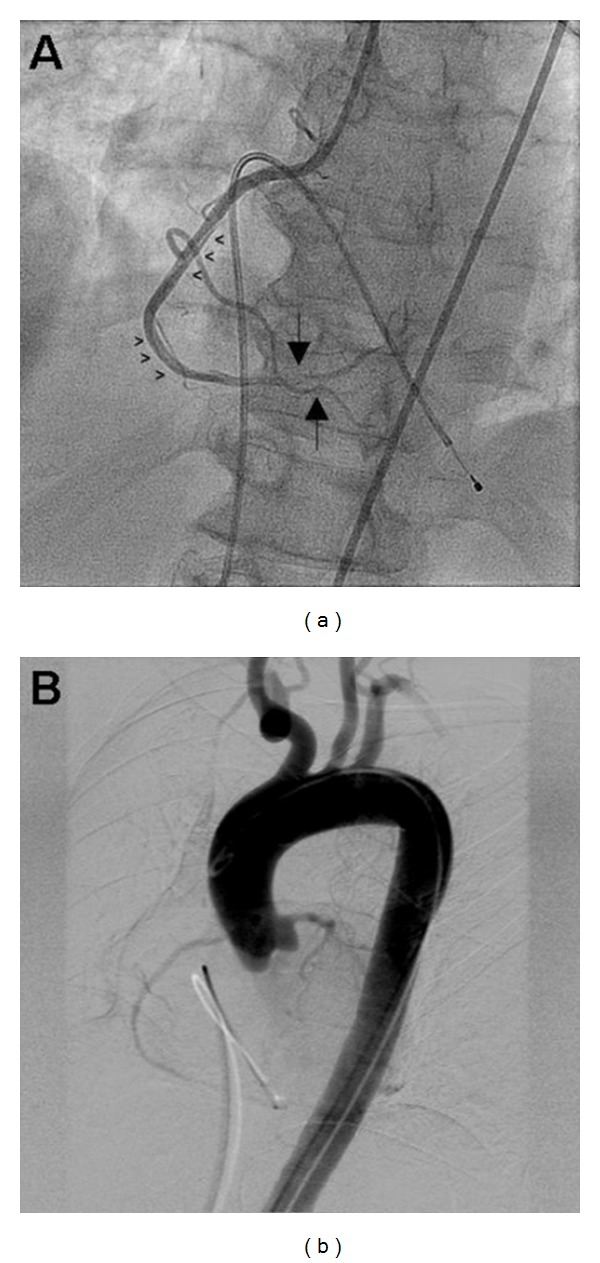
(a) Selective angiography of the right coronary artery (RCA) demonstrating a long dissection extending from the mid-to-distal portions (arrowheads), and thrombotic subtotal occlusion of the posterior descending and posterolateral left ventricular arteries (large arrows). (b) Digitally subtracted aortography showing no evidence of ascending aortic dissection.

**Figure 3:**
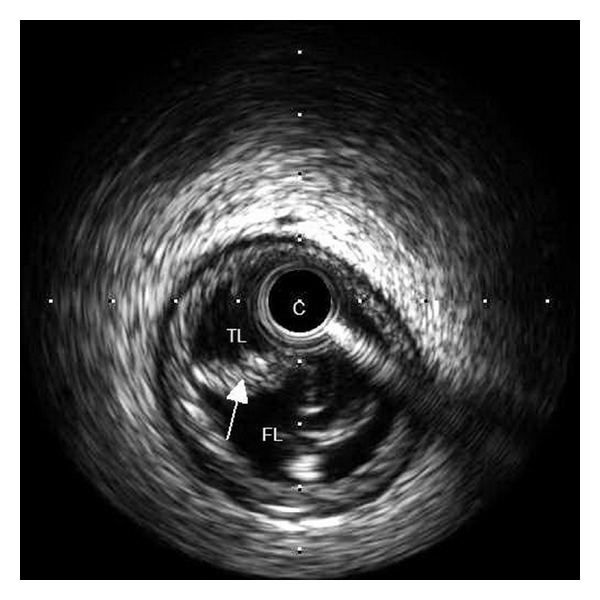
IVUS of the RCA showing evidence of coronary artery dissection (intimal flap marked with white arrow) which delineates the true and false lumens. C: IVUS catheter; FL: false lumen; IVUS: intravascular ultrasound; RCA: right coronary artery; TL: true lumen.

The following day, the patient developed severe chest pain radiating to the shoulders, associated with diaphoresis, lightheadedness, and a brief episode of syncope. Paramedics were called, and the patient was transported to the emergency department. Initial examination revealed a diaphoretic woman with a pulse of 40 beats per minute and a blood pressure of 144/70 mmHg. Notable exam findings included elevated jugular venous pulsations with Cannon A waves. An EKG revealed sinus tachycardia with 3rd-degree A-V block and a slow junctional escape rhythm, inferior ST segment elevation, and lateral ST segment depression. A right-sided EKG was then obtained with similar findings along with ST segment elevation in V4r–V6r suggesting right ventricular involvement (Figure 1). The patient was taken emergently to the cardiac catheterization laboratory, and a temporary transvenous pacemaker wire was inserted. Coronary angiography disclosed a spiral dissection of the mid-right coronary artery (RCA) with thrombus in the posterior descending and posterolateral ventricular arteries (Figure 2(a)) without evidence of ascending aortic dissection on aortography (Figure 2(b)). The extent of the right coronary dissection was further characterized with intravascular ultrasound (Figure 3). The remaining coronary arteries were normal.

Initial abnormal laboratory results included a CKMB of 23.8 ng/mL, a serum troponin T of 0.21 ng/mL, and a platelet count of 84 K/uL. Given the clinical history and the substantial thrombocytopenia, there was a high suspicion for heparin-induced thrombocytopenia; argatroban was therefore utilized for anticoagulation. Catheter-based thrombectomy was performed to alleviate thrombotic occlusion of the distal RCA branches, with restoration of TIMI-3 flow and resolution of the ST elevations and complete heart block. The RCA dissection was managed conservatively without the need for balloon angioplasty or stent placement. The patient was transferred to the cardiac intensive care unit, where follow-up transthoracic echocardiography revealed an ejection fraction of 74% without wall motion abnormalities. A heparin-PF4 antibody test was highly positive, with an optical density of 3.3, corroborating the diagnosis of heparin-induced thrombocytopenia. A comprehensive duplex assessment for vascular thrombosis disclosed thrombus in both cephalic veins. Laboratory evaluation for other hypercoagulable conditions was negative.

The patient recovered quickly and was transitioned to warfarin therapy which was continued for three months as an outpatient. The platelet count returned to baseline. The patient has remained free from any additional cardiac or thromboembolic complications.

## 3. Discussion

While myocardial infarction is relatively common, coronary dissection and heparin-induced thrombocytopenia represent two rare, independent etiologic mechanisms. Clinically significant cardiac injury after chest trauma occurs in up to 15% of patients (ranging from simple benign arrhythmias to lethal conditions); however, coronary artery injury is unusual [1]. Based on autopsy data, the incidence of coronary artery injury after blunt chest trauma is 2% [2]. The mechanism of coronary injury includes intimal tear, dissection, thrombosis, and vasospasm [3]. Coronary artery dissection most commonly affects the left anterior descending artery followed by the right coronary artery and the circumflex [4].

In addition to blunt chest trauma, other causes for coronary dissection include spontaneous dissections due to pregnancy (and the peripartum period), connective tissue disorders, and vasculitides as well as iatrogenic dissections from coronary angiography [5–7]. Dissection typically occurs in the outer tunica media or between the media and the adventitia [6, 8]. In stable patients without evidence of persistent ischemia, conservative management with medical therapy (beta blockers, nitrates, and antiplatelet agents) is reasonable, although endothelial flow-limiting disruption may persist. If conservative therapy fails to alleviate coronary ischemia, percutaneous revascularization or bypass surgery may be required [5, 8].

Heparin-induced thrombocytopenia (HIT) is an immune-mediated prothrombotic disease that may lead to severe thromboembolic complications. In the US, there are 600,000 new cases of HIT annually, of which half will develop venous or arterial thrombosis [9]. HIT is characterized by the formation of antibodies directed against the platelet factor 4 (PF4) complex, with subsequent platelet and vascular endothelial activation [10]. HIT may develop from any type of heparin exposure; however, it occurs less commonly following exposure to low-molecular-weight heparin than to unfractionated heparin. Thrombocytopenia due to HIT may be either relative (50% decrease from baseline) or absolute (less than 150 K/uL), and typically occurs 5–14 days after heparin exposure. Following the development of HIT, there is an associated thromboembolic risk of 5–10% per day after the discontinuation of heparin; this risk of thrombosis may persist for several months after recovery of the platelet count [9]. The clinical diagnosis of HIT may be confirmed using laboratory evaluations. The most common test is the HIT ELISA test which measures antibodies to the PF4 complex. While this test has high sensitivity (90%), it is not very specific, detecting antibodies that do not induce HIT. The C-serotonin release assay (SRA) is the gold standard test with high sensitivity and specificity; however, it is difficult to perform and time consuming. Recent studies have shown that the magnitude of a positive HIT ELISA, expressed as optical density (OD) units, may improve the specificity of the test [11, 12]. When the OD exceeds 1.4, the likelihood of strongly positive SRA was greater than 50%; when the OD is more than 2.0 the likelihood of a strongly positive SRA is nearly 90% [11].

HIT is one of the most prothrombotic states known, with an odds ratio of 37 for thrombosis. Venous thromboembolic complications occur far more commonly than those in the arterial circulation. The site of thrombosis is influenced by multiple factors; areas of vascular injury and endothelial dysfunction are the most likely sites [9]. Although myocardial infarction is a rare complication of HIT, the risk is significantly increased when the integrity of the vascular endothelium has been compromised; this is most often seen in coronary bypass grafts, although disrupted native coronary vessels are also at risk [9, 10, 13]. Acute treatment includes discontinuation of all heparin products, the initiation of a direct thrombin inhibitor such as argatroban, and bridging to warfarin, regardless of clinically-apparent thrombosis [9, 14].

## 4. Conclusion

Both coronary artery dissection and heparin-induced thrombocytopenia are known to cause myocardial infarction; however, this is the first reported case in which both disease states contributed to the pathogenesis in one individual. This patient's acute coronary syndrome likely resulted from the combination of endothelial disruption from coronary artery dissection in the context of chest trauma, compounded by acute thrombosis, coronary occlusion, and subsequent myocardial infarction due to heparin-induced thrombocytopenia. Given the correct clinical context, health care providers should be prepared to evaluate these disease states, since early detection may prevent life-threatening complications.
